# Knowledge and Awareness About Risk Factors, Clinical Manifestations, and Prevention of Thyroid Disorders in the Era of COVID-19 and Their Association With Socioeconomic Status Among the General Population in Riyadh, Saudi Arabia

**DOI:** 10.7759/cureus.48878

**Published:** 2023-11-16

**Authors:** Rayan Abubakker Qutob, Bassam Abdulaziz Alhusaini, Saad Abdullah Alzmamy, Omar Abdulaziz Alfozan, Abdulmalak Abdullah Alsaleh, Fadhah Saud Alhudayris, Lama khalid Alshuaibi, Feras Ahmed Almajed, Abdullah Hussien Alghamdi, Abdullah Alaryni, Yousef Mohammed Alammari, Khalid M Al Harbi, Khalid I AlHussaini, Abdulrahman Mohammed Alanazi, Osamah Ahmad Hakami

**Affiliations:** 1 Department of Internal Medicine, College of Medicine, Imam Mohammad Ibn Saud Islamic University, Riyadh, SAU; 2 Faculty of Medicine, Imam Mohammad Ibn Saud Islamic University, Riyadh, SAU; 3 Department of Internal Medicine, College of medicine, Imam Mohammad Ibn Saud Islamic University, Riyadh, SAU; 4 Department of Internal Medicine, King Abdullah Medical City in Holy Capital (KAMC-HC), Makkah, SAU

**Keywords:** knowledge, covid-19, risk factors, thyroiditis, hyperthyroidism, hypothyroidism, thyroid disorder

## Abstract

Background

Thyroid dysfunction represents the most commonly observed endocrine illness within the population of Saudi Arabia. Thyroid cancer has been recognized as the second most commonly occurring malignant neoplasm among women in Saudi Arabia. Furthermore, there is evidence suggesting that COVID-19 and, to a certain degree, immunization may have an impact on thyroid function. The aim of this study was to evaluate the level of public knowledge, awareness, and attitudes pertaining to the manifestations and risk factors of thyroid disease. Additionally, the study sought to examine the potential role of COVID-19 as a risk factor and explore preventive measures in the context of Riyadh, Saudi Arabia.

Methods

A cross-sectional online survey was conducted targeting the Saudi population living in Riyadh aged 18 years and older. A self-administered questionnaire constructed on Google Forms was distributed to the general population using an online platform. The questionnaire consisted of five sections: demographic data, risk factors for thyroid disorders, clinical manifestations, prevention, and history of thyroid disease. Binary logistic regression analysis was used to identify predictors of better knowledge of thyroid diseases.

Results

Among the 693 participants enrolled, 57.7% were female, and 31.7% were aged between 18 and 25 years. The overall mean knowledge score was 12.2 (SD = 6.57) out of 23 points. Poor knowledge of the risk factors, clinical manifestations, and prevention was observed in 50.4% of the participants. A total of 27.6% had moderate knowledge, and 22.1% had good knowledge levels. Furthermore, only 33.9% of the participants believed that COVID-19 infection was a risk factor. The results of the binary logistic regression analysis revealed that individuals within the age range of 36-45 years, females, and students had a significantly higher level of knowledge compared to other participants (p<0.05).

Conclusion

This study revealed that the general population of Riyadh, Saudi Arabia, lacked adequate knowledge, awareness, and attitudes regarding the risk factors, clinical symptoms, and prevention of thyroid problems. However, middle-aged individuals, females, and those who were enrolled as students showed a higher level of knowledge. Regarding the pathogenesis of COVID-19, it was observed that all participants had a limited understanding and a lack of awareness. Insufficient public awareness may result in misunderstandings, insufficient identification, and potential oversight of COVID-19-infected patients with thyroid dysfunction. Therefore, it is imperative that healthcare authorities intensify their efforts to broaden the dissemination of information throughout the population.

## Introduction

The thyroid gland, the largest endocrine organ, is composed of two lobes situated on the trachea, located inferior to the laryngeal prominence in the cervical region. The thyroid gland is primarily responsible for the secretion of hormones that regulate several physiological processes, such as metabolic rate, cognition, protein synthesis, and the growth and development of children. Additionally, it is responsible for the secretion of calcitonin, an essential hormone involved in the regulation of calcium homeostasis [1]. Both insufficient and excessive levels of iodine have the potential to interfere with the production of thyroid hormones [2,3]. Multiple investigations have indicated a higher prevalence of hypothyroidism compared to hyperthyroidism [4,5]. According to the World Health Organization (WHO), a significant global population of over 190 million individuals is affected by thyroid problems that are associated with iodine deficiency [6]. Hypothyroidism is a commonly occurring medical disorder that has the potential to result in adverse repercussions if left untreated. There are multiple factors contributing to this phenomenon, with one of them being the consequences of post-viral infections, such as COVID-19 [7,8]. The most prevalent etiologies of hyperthyroidism are Graves' disease and toxic nodular goiters [9,10]. Moreover, there is evidence suggesting that COVID-19 infection and, to a certain degree, immunization may have an impact on thyroid function. According to a recent study, it has been found that COVID-19 has the potential to induce thyroid dysfunction, which encompasses subclinical and atypical thyroiditis. It is worth noting that these conditions are generally curable in nature. In addition, it was shown that roughly 7% of patients who participated in a study focusing on individuals who had recovered from SARS exhibited symptoms of hypothyroidism [11]. A separate investigation demonstrated a correlation between COVID-19 and various thyroid disorders, encompassing thyrotoxicosis, subacute thyroiditis (SAT), and non-thyroidal sickness syndrome (NTIS). Furthermore, the injection of COVID-19 vaccinations has been documented to result in thyroid problems. Subacute thyroiditis emerged as the prevailing thyroid disorder associated with the COVID-19 vaccination, as evidenced by its high incidence rate [12]. Thyroiditis that is linked to COVID-19 can be categorized into two distinct types. One type of thyroiditis that occurs during the early stages of COVID-19 infection is characterized as "destructive" and is typically observed in males. Lymphopenia is a commonly observed condition that is often asymptomatic. Another complication that might occur is subacute thyroiditis, which typically affects women who are experiencing symptoms. It typically arises around one month after the COVID-19 infection and is characterized by mild leukocytosis. According to available data, thyroid dysfunction stands as the most commonly observed endocrine condition within the population of Saudi Arabia. Furthermore, it has been observed that thyroid cancer exhibits a significantly high frequency within the Hail region, making it the second most commonly occurring malignant neoplasm among women in Saudi Arabia [13]. A survey conducted in 2019 and subsequently published revealed a notable lack of awareness regarding thyroid problems among the Saudi population. There were two research studies conducted in Riyadh [14,15] and one study each in Al-Majmaah [16], Tabuk [17], and Makkah [18], which revealed that a significant proportion of participants exhibited insufficient levels of knowledge pertaining to thyroid problems. To the best of our current understanding, there has been no prior investigation conducted in Saudi Arabia that has evaluated the general public's knowledge and awareness regarding COVID-19 as a potential risk element for thyroid dysfunction. Furthermore, previous research has independently examined the understanding and consciousness regarding the symptoms, factors contributing to, and methods of preventing thyroid disease in Riyadh. In this study, we integrated three components to conduct a comprehensive evaluation of the general population's knowledge and awareness pertaining to the manifestations of thyroid disease, risk factors (including inquiries regarding COVID-19 infections associated with thyroid dysfunction), and preventive measures. Our goal was to enhance awareness-raising initiatives aimed at addressing this matter.

## Materials and methods

Study design

This cross-sectional survey study was conducted between September 2022 and January 2023 on adult populations living in Riyadh, Saudi Arabia.

Sampling technique

Data was collected using a convenience sample technique, following the acquisition of informed consent and the declaration of the veracity of the submitted information. The survey was created using the Google Forms platform and disseminated via various social media channels (including Facebook, Snapchat, Twitter, and WhatsApp) in order to gather data from eligible participants who met the specified inclusion criteria. These criteria encompassed individuals residing in Riyadh, Saudi Arabia, both Saudi and non-Saudi, who were 18 years of age or older. Individuals below the age of 18 and those who declined to participate were excluded from the study. At the onset of the questionnaire, participants were presented with informed consent and a certification of authenticity.

Questionnaire tool

The present investigation employed a questionnaire tool that had been previously validated [19]. The survey utilized in this research comprised a total of 36 questions, which were then categorized into five distinct categories. The initial set of questions (comprising seven questions) pertained to demographic information, specifically encompassing nationality, gender, age, social standing, educational background, occupation, and monthly income. The second section focused on understanding the risk factors associated with thyroid problems. It comprised a total of 13 questions, with six specifically addressing the impact of COVID-19 on the thyroid gland. The third section encompassed an examination of the clinical symptoms associated with thyroid disorders, consisting of a total of seven questions. The fourth section pertained to the understanding of preventive measures for thyroid illness, consisting of three questions. The fifth section focused on the individual's medical history and familial background related to thyroid disease, encompassing six questions.

A questionnaire consisting of 23 items was used to evaluate the collective knowledge of the general public regarding the risk factors, clinical symptoms, and prevention strategies associated with thyroid problems. The respondents were required to select either "yes" (coded as 1) or "no/I don't know" (coded as 0) as their answer alternatives. The cumulative knowledge score was determined by summing the responses to all 23 items, resulting in a score ranging from 0 to 23. A higher score indicates a greater understanding of risk factors, clinical symptoms, and prevention strategies related to thyroid problems. Participants were classified as possessing inadequate knowledge if their scores fell below the 50% threshold, with 50% and 75% as the respective cut-off criteria for determining knowledge levels. Moderate knowledge was defined as a score within the range of 50% to 75%, while good knowledge was classified as a score above 75%.

Ethical approval

This study was approved by the Institutional Review Board at Al-Imam Muhammad Ibn Saud Islamic University (Project number 322/2022).

Statistical analysis

Data were analyzed using IBM Corp. Released 2021. IBM SPSS Statistics for Windows, Version 29.0. Armonk, NY: IBM Corp. For the descriptive analysis, the mean (standard deviation (SD)) was used for metric variables, while frequencies and proportions (%) were used for categorical variables. The normality test was performed using the Shapiro-Wilk and Kolmogorov-Smirnov tests. The knowledge score followed the normal distribution. Differences in knowledge scores according to sociodemographic characteristics and previous history of thyroid disorders were assessed using the Student’s t-test and ANOVA. Binary logistic regression analysis was used to identify predictors of better knowledge of thyroid diseases. Values were considered significant at a p-value of less than 0.05.

## Results

Participants’ demographic characteristics

A total of 693 participants were included in the present study. Table 1 presents a comprehensive summary of the sociodemographic features exhibited by the participants. The study revealed that a significant proportion of the participants, specifically 31.7%, fell within the age range of 18 to 25 years. Furthermore the majority of the participants, accounting for 57.7%, identified as female. The majority of participants were of Saudi nationality, with 47.8% reporting their marital status as single. A majority of the participants, specifically 51.2%, possessed a bachelor's degree, whereas 30% of them were engaged in employment within the public sector. Furthermore, it was found that a majority of individuals, specifically 54%, had a monthly income of less than 5,000 Saudi Arabia riyals (SAR).

**Table 1:** Participants’ socio-demographic characteristics SAR: Saudi Arabia riyal

Study variables	N (%)
Age group	
18 - 25 years	220 (31.7%)
26 - 35 years	198 (28.6%)
36 - 45 years	134 (19.3%)
46 - 55 years	98 (14.1%)
56 - 65 years	37 (05.3%)
>65 years	06 (0.90%)
Gender	
Male	293 (42.3%)
Female	400 (57.7%)
Nationality	
Saudi	561 (81.0%)
Non-Saudi	132 (19.0%)
Marital status	
Single	331 (47.8%)
Married	302 (43.6%)
Divorced	44 (06.3%)
Widowed	16 (02.3%)
Educational level	
Below high school	63 (09.1%)
High school	167 (24.1%)
Diploma	49 (07.1%)
Bachelor's degree	355 (51.2%)
Postgraduate degree	59 (08.5%)
Employment status	
Public sector employee	208 (30.0%)
Private sector employee	119 (17.2%)
Self-employed	37 (05.3%)
Unemployed	109 (15.7%)
Retired	36 (05.2%)
Student	184 (26.6%)
Monthly income (SAR)	
<5,000	374 (54.0%)
5,000 – 10,000	143 (20.6%)
11,000 – 15,000	92 (13.3%)
16,000 – 20,000	51 (07.4%)
>20,000	33 (04.8%)

Previous medical history and family history of thyroid disorder

According to the data presented in Table 2, a total of 19.3% of the participants were found to have been diagnosed with thyroid disease. Hypothyroidism was found to be the most prevalent thyroid condition, accounting for 50.7% of the cases. A prevalence of 39.7% has been documented for the familial occurrence of thyroid problems, with hypothyroidism being the most often reported condition, accounting for 60% of the cases. The percentage of individuals who received thyroid gland screening was 33.9%, whereas 48.9% underwent standard checkups.

**Table 2:** Previous medical history and family history of thyroid disorders

Study variables	N (%)
Have you ever had thyroid disease?	
Yes	134 (19.3%)
No	559 (80.7%)
Which type of thyroid disorder do you have? ^(n=134)^	
Hypothyroidism	68 (50.7%)
Hyperthyroidism	34 (25.4%)
Thyroid nodules	09 (06.7%)
Thyroid cancer	05 (03.7%)
I don't know	18 (13.4%)
Have anyone in your family been diagnosed with a thyroid disorder?	
Yes	275 (39.7%)
No	347 (50.1%)
I don’t know	71 (10.2%)
Which type of thyroid disorder? ^(n=275)^	
Hypothyroidism	165 (60.0%)
Hyperthyroidism	46 (16.7%)
Thyroid nodules	09 (03.3%)
Thyroid cancer	18 (06.5%)
I don't know	37 (13.5%)
Have you ever done any thyroid gland investigations?	
Yes	235 (33.9%)
No	402 (58.0%)
I don’t know	56 (08.1%)
What was the reason for the investigation of thyroid function? ^(n=235)^	
Routine check	115 (48.9%)
Doctor suggestion	52 (22.1%)
Symptoms of thyroid disease	44 (18.7%)
Neck swelling	07 (03.0%)
I don't know	17 (07.2%)

Knowledge about risk factors, clinical manifestations, and prevention of thyroid disorders

The analysis of participants' understanding of risk factors, clinical manifestations, and prevention of thyroid disorders (Table 3) revealed that they possessed knowledge regarding the prevalence of various risk factors.

**Table 3:** Assessment of knowledge about risk factors, clinical manifestations, and prevention of thyroid disorders in the era of COVID-19 SD: Standard deviation

Knowledge regarding risk factors of thyroid disorders	Yes (%)
Do you think females are more at risk of having thyroid diseases?	439 (63.3%)
Do you think radiation exposure is a risk factor for thyroid diseases?	435 (62.8%)
Do you think smoking is a risk factor for thyroid diseases?	407 (58.7%)
Do you think insufficient or excess iodine intake is a risk factor for thyroid diseases?	406 (58.6%)
Do you think pregnancy and the postpartum period are risk factors for thyroid diseases?	327 (47.2%)
Do you think lithium intake is a risk factor for thyroid diseases?	268 (38.7%)
Do you think the viral upper respiratory infection is a risk factor for thyroid diseases?	254 (36.7%)
Do you think the medication Amiodarone (Known commercially as Pacerone, CORDARONE, ADVADARONE, SEDACORON) is a risk factor for thyroid diseases?	204 (29.4%)
Knowledge regarding COVID-19 infection as a risk factor for thyroid disease	
Do you think COVID-19 infection can result in transient thyroid dysfunction?	324 (46.8%)
Do you think COVID-19 can infect the thyroid gland itself causing thyroiditis?	256 (36.9%)
Do you think COVID-19 infection can result in permanent thyroid dysfunction?	251 (36.2%)
Do you think COVID-19 can trigger an autoimmune response against the thyroid gland resulting in thyroid diseases?	247 (35.6%)
Do you think COVID-19 infection is a risk factor for thyroid diseases?	235 (33.9%)
Knowledge about the clinical manifestations of thyroid disorders	
Do you think fatigue can be a symptom of thyroid disease?	537 (77.5%)
Do you think the neck lump can be a sign of thyroid disease?	502 (72.4%)
Feeling cold and weight gain is a common symptom of having hypothyroidism	494 (71.3%)
Feeling hot and weight loss is a common symptom of hyperthyroidism	460 (66.4%)
Do you think Bulging eyes can be a sign of thyroid disease?	433 (62.5%)
Do you think skin and nail changes or hair loss can be signs of thyroid diseases?	343 (49.5%)
Do you think diarrhea, constipation, or stomachache can be symptoms of thyroid diseases?	304 (43.9%)
Knowledge about the prevention of thyroid disorders	
Do you think early thyroid function tests can prevent the complication of thyroid diseases?	552 (79.7%)
Do you think a well-balanced diet is essential to prevent thyroid diseases?	516 (74.5%)
Do you think being away from Soy food is one of the preventive ways for thyroid diseases in women?	269 (38.8%)
Total knowledge score (mean ± SD)	12.2 ± 6.57
Level of knowledge	
Poor	349 (50.4%)
Moderate	191 (27.6%)
Good	153 (22.1%)

The most widely recognized risk factor for thyroid disorder was identified as being female, with a recognition rate of 63.3%. In relation to the correlation between knowledge of COVID-19 infection and the risk of thyroid disease, it was shown that a majority of participants had a limited understanding of this connection. In relation to the understanding of thyroid disease prevention, it was observed that a majority of the participants held the belief that early thyroid screening is imperative in order to mitigate the potential complications associated with thyroid diseases. Additionally, a significant proportion of respondents (approximately 74.5%) expressed the view that maintaining a well-balanced diet plays a crucial role in preventing thyroid dysfunction.

The average knowledge score was found to be 12.2 (SD: 6.8). The distribution of knowledge levels was as follows: 50.4% had poor knowledge, 27.6% had moderate knowledge, and 22.1% had strong knowledge. When examining the variations in knowledge scores with respect to participants' sociodemographic characteristics and previous history of thyroid disease (as shown in Table 4), it was observed that a higher knowledge score was more prevalent among female individuals (p=0.013), Saudi nationals (p=0.002), employed individuals or students (p=0.002), individuals with a monthly income below 5,000 SAR (p=0.013), individuals with a previous diagnosis of thyroid disease (p=0.001), and individuals who had undergone thyroid gland investigation (p<0.001).

**Table 4:** Differences in the mean score of knowledge according to participants’ socio-demographic characteristics and previous history of thyroid disorders ** Significant at p<0.05 level; SAR: Saudi Arabia riyal

Factor	Knowledge Score (23) Mean ± SD	P-value
Age group		
≤35 years	11.9 ± 6.3	0.293
>35 years	12.6 ± 7.0	
Gender		
Male	11.6 ± 6.9	0.013 **
Female	12.7 ± 6.3	
Nationality		
Saudi	12.6 ± 6.7	0.002 **
Non-Saudi	10.5 ± 5.5	
Marital status		
Unmarried	11.9 ± 6.6	0.196
Married	12.7 ± 6.6	
Educational level		
Diploma or below	12.7 ± 7.3	0.237
Bachelor or higher	11.9 ± 6.0	
Employment status		
Employed/Student	12.6 ± 6.8	0.002 **
Unemployed	10.7 ± 5.6	
Monthly income (SAR)		
<5,000	12.7 ± 6.7	0.013 **
≥5,000	11.6 ± 6.3	
Previous history of thyroid disease		
Yes	14.2 ± 6.6	0.001 **
No	11.7 ± 6.5	
Family history of thyroid disease		
Yes	12.5 ± 5.5	0.651
No	12.4 ± 7.1	
Ever done thyroid gland investigations		
Yes	13.5 ± 5.7	<0.001 **
No	11.6 ± 6.8	

The results of the binary logistic regression analysis revealed that individuals within the age range of 36-45 years, females, and students had a significantly higher level of knowledge compared to other participants (p<0.05), as shown in Table 5.

**Table 5:** Binary logistic regression analysis. SAR: Saudi Arabia riyal, *p<0.05; **p<0.01; ***p<0.001

Study variables	Odds ratio of having better knowledge score (95% confidence interval)
Age group	
18 - 25 years (Reference category)	1.00
26 - 35 years	0.99 (0.67-1.46)
36 - 45 years	1.97 (1.28-3.06)**
46 - 55 years	0.72 (0.44-1.17)
56 - 65 years	0.30 (0.13-0.72)**
>65 years	-
Gender	
Male (Reference category)	1.00
Female	1.37 (1.01-1.86)*
Marital status	
Single (Reference category)	1.00
Married	0.96 (0.70-1.32)
Divorced	0.56 (0.29-1.10)
Widowed	0.94 (0.34-2.58)
Educational level	
Below high school (Reference category)	1.00
High school	0.96 (0.53-1.71)
Diploma	0.88 (0.42-1.87)
Bachelor's degree	0.95 (0.56-1.63)
Postgraduate degree	0.65 (0.31-1.34)
Employment status	
Public sector employee (Reference category)	1.00
Private sector employee	0.39 (0.25-0.62)***
Self-employed	0.33 (0.16-0.70)**
Unemployed	0.40 (0.25-0.65)***
Retired	0.11 (0.04-0.30)***
Student	0.56 (0.37-0.83)**
Monthly income (SAR)	
<5,000 (Reference category)	1.00
5,000 – 10,000	0.91 (0.62-1.34)
11,000 – 15,000	0.80 (0.51-1.27)
16,000 – 20,000	0.55 (0.29-1.01)
>20,000	0.20 (0.07-0.52)***

## Discussion

The present study investigated the extent of knowledge among the general public pertaining to risk factors, clinical symptoms, and preventive measures associated with thyroid diseases. The results of this study indicate that there is a lack of understanding among people regarding thyroid problems. Roughly 50.4% of the participants in our study were classified as possessing inadequate knowledge (a score below 50%), while 27.6% were deemed to have moderate knowledge (a score within the range of 50% to 75%), and only 22.1% were identified as having good knowledge (a score above 75%). The mean score for knowledge attainment was calculated to be 12.2 (SD: 6.6), based on a total of 23 possible points. This observation aligns with the results reported by Alhazmi et al. [18]. Based on prior studies, it was found that a significant proportion of patients, specifically 69.5%, demonstrated a limited understanding of hypothyroidism. The findings of Alyahya et al. [19] have provided confirmation that a significant proportion of residents in the Eastern Province exhibited inadequate levels of knowledge, with 44.7% falling into this category. A survey conducted among pregnant women in India [20] revealed that the vast majority (90%) exhibited insufficient information regarding thyroid problems. Only a small proportion (10%) indicated a moderate level of knowledge, while none of the participants were classified as having a high level of knowledge. These findings indicate lower knowledge levels compared to the reports presented in our study. In contrast, a survey conducted in Taif City [1] revealed that the general population exhibited a good level of knowledge. Insufficient knowledge within the community might lead to the failure to identify and diagnose diseases. Hence, it is imperative to emphasize the significance of ongoing endeavors in health education to enhance community knowledge pertaining to fundamental aspects of thyroid disorders.

In relation to the data pertaining to the association between awareness of COVID-19 infection and the risk of thyroid disease, it was observed that a majority of the participants exhibited inadequate understanding of the correlation between COVID-19 infection and thyroid disorder. Insufficient research has been conducted on the collective understanding of COVID-19 within the general population and its potential correlation with thyroid disease. Regrettably, only 33.9% of the participants demonstrated awareness regarding the association between COVID-19 and thyroid disease as a risk factor. Furthermore, a significant proportion of individuals (46.8%) demonstrated awareness regarding the potential of COVID-19 infection to result in transitory health complications (36.2%). This finding aligns with the research conducted by Lisco et al., where it was observed that COVID-19 has the capacity to induce transient and manageable thyroid dysfunction [21]. Twelve individuals, accounting for 36.2% of the sample, exhibited permanent thyroid dysfunction. The limited awareness within the population may result in misunderstandings concerning the correlation between thyroid disease and COVID-19, perhaps resulting in delayed diagnoses or overlooked instances [22-24].

Knowledge of thyroid diseases is influenced by several demographic characteristics. The findings of our study suggest that several characteristics, namely female sex, Saudi nationality, employment or student status, lower economic income, past diagnosis of thyroid disease, and undergoing thyroid gland screening, significantly influenced knowledge scores. In addition, binary logistic regression analysis revealed that individuals within the age range of 36-45 years, females, and students had a significantly higher level of knowledge compared to other participants (p<0.05). The results of this study were consistent with the findings published by Alyahya et al. [19]. They observed that female students residing in Al Ahsa who had received a diagnosis of a thyroid ailment, possessed a familial predisposition to the disease, and had undergone thyroid gland examination demonstrated a higher level of awareness of thyroid diseases compared to the general population. In accordance with these findings, Alqahtani [16] observed a notable correlation between knowledge of thyroid diseases and variables such as age, sex, and education [16]. Similarly, a survey conducted among Nepalese women [25] identified university education, working status, urban residence, and family history of thyroid disorder as significant factors associated with increased knowledge. In contrast to these reports, Alotaibi et al. (2019) found no statistically significant relationship between demographic variables, such as economic status and education level, and the level of knowledge [15]. Similarly, Almuzaini et al. (2018) and Alhawiti et al. (2022) also observed that age, sex, education, and occupation were not significant factors influencing the level of knowledge about the disease [17,26]. The potential discrepancies in these findings could perhaps be attributed to variations in regional configurations and different questionnaire items being utilized, although it is plausible that additional elements may have been introduced. Therefore, additional research is required to determine the actual impact of demographic factors on individuals' understanding of thyroid diseases.

The inadequate knowledge of our population can be attributed to the complexities surrounding the factual aspects pertaining to the ailment. For example, a lack of sufficient understanding regarding many risk factors associated with thyroid diseases can be observed. These risk factors include pregnancy and the postpartum period (47.2%), use of lithium (38.7%), upper respiratory infection (36.7%), and the use of amiodarone medicine (29.7%). The participants' understanding of the correlation between COVID-19 infection and thyroid dysfunction was determined to be less than ideal. The negative perceptions observed in this study align with the results reported by Alotaibi and Almousa [14, 15]. Consequently, it was shown that only 17% of individuals demonstrated the ability to recognize disruptions in the menstrual cycle, repeated miscarriages, and stillbirths as potential indicators of thyroid issues. Furthermore, only 30.3% of individuals possessed knowledge regarding the association between psychological conditions, such as depression, lack of concentration, mood swings, confusion, and anxiety, and their relevance as risk factors. Similarly, only 27.4% of participants were aware of the fact that neck and joint pain, fatigue, and weight loss were also considered risk factors. Additionally, only 25.3% of respondents believed that constipation and/or diarrhea, voice changes, and swelling of the neck were associated with an increased risk of thyroid diseases.

Nevertheless, despite these limitations, our study participants showed improved knowledge regarding clinical presentations and preventive measures for thyroid problems. Specifically, a significant proportion of subjects exhibited awareness of symptoms such as fatigue (77.5%), neck lumps (72.4%), feeling cold and weight increase (71.3%), feeling hot and weight loss (66.4%), and bulging eyes (62.5%). Furthermore, a significant majority of our study participants showed knowledge of effective strategies for mitigating the risk of thyroid problems. Specifically, 79.7% of participants were familiar with the importance of thyroid screening tests, while 74.5% recognized the significance of maintaining a well-balanced diet. The prevalent symptoms of thyroid illnesses, as reported by 43.9% of the participants, encompassed solely diarrhea, constipation, and stomachaches. Additionally, a notable proportion of women (38.8%) were unaware of the potential preventive measure of avoiding a soy diet to mitigate the risk of thyroid disease. Alhawiti et al. have also documented these circumstances [17]. The majority of participants acknowledged the clinical presentation of hypothyroidism, with a specific emphasis on weight gain (76%), as well as lethargy and sleepiness (74.9%). Furthermore, a significant majority of participants (90.4%) demonstrated awareness of the specific individuals that are most vulnerable to the disease. Insufficient documentation exists regarding the functionality of the thyroid gland, the etiology of thyroid hormonal imbalances, and manifestations of hyperthyroidism.

The study is limited by the utilization of convenience sampling techniques and the reliance on self-reported data from participants, both of which are commonly associated with response and recall biases, respectively. Furthermore, the study was constrained to the demographic residing specifically in Riyadh, Saudi Arabia, hence potentially lacking generalizability to the broader Saudi community. In order to maximize the generalizability of the findings, future studies ought to take into account larger sample sizes and incorporate a more diverse population.

## Conclusions

Overall, this study revealed an inadequate level of knowledge, awareness, and attitude pertaining to risk factors, clinical symptoms, and prevention of thyroid problems among the general populace residing in Riyadh, Saudi Arabia. However, a higher level of knowledge was observed among middle-aged Saudi women and those who were enrolled as students. We observed that all participants had a limited understanding and lack of awareness regarding the pathogenesis of COVID-19. Therefore, it is imperative for healthcare authorities to intensify their efforts in order to enhance the scope of information transmission across the population. There is a need for more health education efforts aimed at improving the general understanding and attitudes around thyroid illnesses, with a particular focus on addressing COVID-19 as a potential risk factor.
